# Investigating ChatGPT-mediated mind mapping to facilitate EFL learners’ reading comprehension

**DOI:** 10.1371/journal.pone.0336185

**Published:** 2026-05-18

**Authors:** Naji Alyami

**Affiliations:** Department of English, College of Languages and Translation & Sharia, Educational and Humanitarian Research Center, Najran University, Najran, Saudi Arabia; Golestan University, IRAN, ISLAMIC REPUBLIC OF

## Abstract

Mastering Reading Comprehension (RC) is a significant challenge for English as a Foreign Language (EFL) learners. This quasi-experimental mixed-methods study examined the potential effectiveness of a ChatGPT-mediated mind mapping technique in enhancing RC among EFL students at a public university in Saudi Arabia. Sixty male preparatory-year students were assigned to two groups: an experimental group (n = 30), which took part in a 10-week intervention in ChatGPT-mediated mind mapping, and a control group (n = 30), which was taught mind mapping through conventional methods. Data were gathered through pre- and post-tests of RC together with semi-structured interviews. Post-test RC scores were significantly higher in the experimental group than in the control group (U = 180.00, p < .001), with a medium-to-large effect size (r = 0.52). The qualitative data showed that students found the technique useful for breaking down complex ideas and for making the relationships between concepts in the text visible. At the same time, they reported difficulties with the accuracy of the AI output, with comprehending dense content, and with technology access. Read through the lenses of Sociocultural Theory and Cognitive Load Theory, the findings suggest that ChatGPT-mediated mind mapping can serve as a useful pedagogical tool for supporting RC. Teachers are therefore encouraged to incorporate the technique into their instructional practice while offering the support needed to address the challenges identified here.

## Introduction

Reading comprehension in English poses real difficulties for EFL learners, who can often decode individual words yet struggle to piece together the meaning that emerges when those words are combined into a coherent idea [1]. RC is itself an active process in which readers interpret the text [2], draw out key information [3], identify the author’s intended message [4], and draw on prior knowledge to construct meaning [5].

Generative AI tools such as ChatGPT can support EFL learners’ RC when paired with mind mapping. In this role, ChatGPT contributes to EFL reading instruction by making the comprehension of passages more interactive and visually grounded, helping learners organize, analyze, and work through texts in ways that ultimately strengthen their RC [6].

A growing body of research on electronic mind mapping reports positive effects on RC across a range of EFL contexts [6–22]. However, using mind mapping for RC poses challenges, such as difficulties in idea selection, fostering creativity, and determining optimal use in individual or collaborative settings [23]. Not all text types lend themselves equally well to the technique, and its overall effectiveness can also be undermined by uneven time management, difficulties in collaboration, and usability issues with digital tools [22,24].

While extensive literature addresses reading strategy instruction globally, empirical research on utilizing generative artificial intelligence to train strategies for Arab university students learning EFL RC is very limited. The present study examines how ChatGPT-mediated mind mapping shapes EFL readers’ comprehension of reading passages. It proceeds from the assumption that, by prompting learners to verify the accuracy of the mind maps ChatGPT generates, the technique can deepen their understanding of the texts they read. Additionally, the study explores the obstacles that EFL readers encounter when using GPT for creating mind maps of reading passages. This study is significant for three key reasons: it emphasizes the role of ChatGPT-mediated mind mapping in expanding learners’ cognitive capacity; it contributes to existing research by introducing a structured online training program; and it underscores the importance of creating an inclusive, user-friendly learning environment. The study is guided by two research questions: (1) Does ChatGPT-mediated mind mapping improve EFL readers’ RC? (2) What are EFL readers’ learning experiences of utilizing the GPT-mediated mind mapping technique to comprehend reading passages?

## Theoretical framework

According to Buzan [25], mind mapping is a potent graphical technique that serves as a universal tool for unleashing the brain’s potential. Mind mapping is a diagrammatic method used to visually represent words, ideas, tasks, or other information. It typically arranges these elements radially around a central keyword or idea [26]. Mind mapping is a tool that aids in memory recall and is recognized for its ability to enhance students’ English proficiency. It serves as a technique that facilitates effective learning by visually organizing information. This method supports constructive language lessons by fostering creativity and facilitating individualized learning processes. Mind mapping can be implemented using the pen and paper tradition or through digital platforms [25]. Olivia [27] outlines several benefits of mind mapping, including enhancing students’ ability to focus on and recall information, fostering creativity, succinctly summarizing course material, facilitating high academic achievement, promoting enjoyable learning experiences, and encouraging the collaboration of both hemispheres of the brain.

Mind mapping is a visual note-taking technique that combines words, images, colors, and symbols in a hierarchical or tree-like layout, with ideas branching outward into subtopics [28]. The format is especially helpful for struggling readers, since it makes the relative weight of different points and the links between facts immediately visible. As a tool for learning and critical thinking, a mind map offers a framework for examining the different dimensions of a narrative: the sequence of events, the main points, cause-and-effect relationships, and the connections among ideas. Students can use mind maps to revisit and clarify their thinking, and in doing so arrive at a deeper understanding of a story [29].

The present study is grounded primarily in Vygotsky’s [30] Sociocultural Theory and Sweller’s [31] Cognitive Load Theory, which together offer a coherent explanation for how GPT-mediated mind mapping may support RC. These two theories were selected because they are directly aligned with the intervention design and help explain both the mediating role of ChatGPT and the cognitive benefits of organizing reading passages visually.

### Vygotsky’s sociocultural theory

According to Vygotsky [30], learning develops through interaction with mediating tools and more capable others. In the present study, ChatGPT functions as a mediating scaffold that supports learners while they analyze reading passages and construct mind maps. ChatGPT does not replace the learner but supports them in locating the main idea, supporting details, and additional information, and in organizing these into a structured representation. Such scaffolding matters most when students are not yet able to carry out these tasks on their own. In this respect, ChatGPT can be understood as operating within the learner’s Zone of Proximal Development, where guided assistance enables the completion of comprehension tasks that would otherwise lie just beyond reach.

This theoretical lens is particularly relevant because the intervention was not based on passive acceptance of AI output. Instead, students were trained to co-construct mind maps with ChatGPT by reading the passage first, entering structured prompts, reviewing the generated output, and then validating it against the original text. This process positions ChatGPT as a supportive mediator in learning rather than a source of ready-made answers. The instructional value of the tool, therefore, lies in its ability to guide learners’ thinking and help them engage more actively with reading passages.

### Cognitive load theory

The second theoretical foundation of the study is Cognitive Load Theory [31]. RC in a foreign language often places a high burden on working memory because learners must simultaneously process vocabulary, sentence structure, and the relationships among ideas. When information is presented in a long linear form, students may struggle to retain and organize it efficiently. Mind mapping can help reduce this burden by externalizing the structure of the text into a visual format. By showing the relationships among ideas clearly, mind maps may reduce extraneous cognitive load and allow learners to focus more effectively on comprehension.

In the context of the present study, GPT-mediated mind mapping may reduce cognitive load in two ways. First, it helps students reorganize textual information into manageable units, making it easier to distinguish central ideas from secondary details. Second, the validation stage asks students to compare the AI-generated map with the original passage, drawing their attention back to both meaning and organization. GPT, on this view, does more than produce an output: it helps learners engage with the text in a more systematic and efficient way.

Taken together, these two theories provide a focused conceptual model for the intervention. Sociocultural Theory explains how ChatGPT functions as a scaffold and mediating tool during mind-map construction, while Cognitive Load Theory explains why organizing information visually may support RC by reducing unnecessary mental burden. Within this model, students’ validation of ChatGPT output is an essential step because it requires them to review, refine, and confirm the meaning of the passage rather than accept the output automatically. Thus, the theoretical framework of the study is not based on multiple separate theories, but on a coherent explanation of how guided mediation and reduced cognitive load work together to support EFL learners’ RC through GPT-mediated mind mapping.

### Previous studies

A number of studies have examined how different mind mapping techniques affect RC across a variety of educational settings. Working with ChatGPT-mediated mind mapping for collaborative brainstorming, Duong [6] reported a significant correlation between students’ grasp of the method and their ability to generate and organize ideas. In the Thai context, Chaichompoo [13] found that electronic mind mapping improved both RC and summarization among English majors. Comparable gains have been documented by Mohaidat [17] with Jordanian students and by Almelhi [9] with EFL college learners. In Indonesian university classrooms, Andoko et al. [12] and Pinandito et al. [20] point to kit-build concept mapping as an effective support for English RC when it is used in collaborative learning. Work by Hamid [15], Alomari [10], and Alomari and Alhorani [11] likewise documents the benefits of digital mind mapping for RC across different educational contexts, while Adel Baghagho et al. [7] report gains on several dimensions of RC among Egyptian preparatory school students. Other studies broaden the picture: Hazaymeh and Alomery [16] show that visual mind mapping strategies enhance critical thinking and reading proficiency among English language learners; Ghorbani Shemshadsara et al. [14] find that computer-based instruction with mind mapping tools strengthens both text-structure awareness and RC at the undergraduate level; and Rittichai and Torat [21] demonstrate the value of mind mapping delivered through platforms such as Edmodo for English reading proficiency. Similar improvements in RC have been observed with electronic mind mapping applications in Novitasari’s [19] work with college students and Monliang’s [18] with high school students. Finally, Aljaser [8] and Yan and Kim [22] report positive effects of electronic mind mapping on academic achievement and on schema strategy instruction, reinforcing the broader role of mind mapping in supporting RC across educational settings.

Recent scholarship on AI in EFL education further indicates that AI-supported instruction can strengthen learners’ higher-order thinking, classroom engagement, and openness to adopting new learning tools. For example, Liu and Wang [32] found that an AI-based instructional intervention in English literature classes was associated with significant gains in EFL learners’ critical thinking. In addition, Wu et al. [33] showed that EFL learners’ intention to use AI is strongly shaped by perceived ease of use and positive attitudes toward AI. From an affective perspective, Derakhshan and Park [34] reported that AI-mediated instruction may increase positive achievement emotions while reducing negative ones. Read together, these studies indicate that AI-supported learning can shape more than performance alone; it also touches on learners’ cognitive engagement, their emotional experience of learning, and their readiness to adopt educational technology.

Using mind mapping as a teaching tool for RC also comes with difficulties for students, particularly around selecting which ideas to foreground and bringing enough creativity to the task. While recognized for its benefits, concerns persist regarding its optimal use—whether individually or in collaborative settings [23]. Kepirianto et al. [24] argue that not all types of texts are suitable for mind mapping, especially when combined with collaborative methods like Think-Pair-Share, as effective comprehension often requires sufficient background knowledge. Yan and Kim [22] highlight additional obstacles such as uneven time management, difficulties in collaboration, and usability issues with digital tools, which detract from the effectiveness of mind mapping instruction in reading education.

## Methods

**Fig 1:**
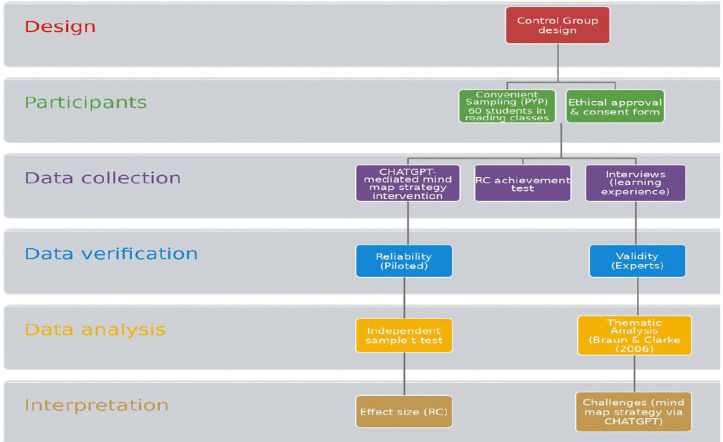
Flow chart of the study procedures.

The current study followed the quasi-experimental mixed method approach with a control design. The study adopted a quasi-experimental, mixed-methods design with a control group. Rather than relying on random assignment, the design compared two groups: an experimental group, which received the intervention, and a control group, which did not. This approach is well suited to evaluating the teaching strategies used with the experimental group while taking pre-existing differences between the two groups into account. In educational settings where random assignment is difficult for logistical or ethical reasons, a design of this kind offers a realistic way to gauge the impact of an intervention. Combining quantitative evidence—such as the reading assessment results—with qualitative evidence drawn from student comments and observations, the mixed-methods component provided a fuller picture of both the intervention’s effect and the students’ experience of it [35]. Two groups (control and experimental) of EFL students enrolled in level one reading class composed the sample of the study. The data collection included a pre-and post-test and a semi-structured interview. Fig 1 shows the flow of study.

### Participants

The study was carried out at a university in the southwestern region of Saudi Arabia, with participants drawn from the Preparatory Year Program. This program plays a pivotal role for science-stream students in the Saudi system, bridging high school and specialized university pathways such as medicine, engineering, computer science, pharmacy, and nursing. Its curriculum is designed to meet university entry requirements and delivers intensive coursework in computer science, mathematics, communication skills, and English. Additionally, the program provides psychological preparation through counseling sessions to help students adjust to university life. In the Preparatory Year, classes are gender-segregated, with male teachers instructing male students and female teachers instructing female students. Due to communication difficulties with the female section, the study specifically targeted the male section.

The study examined the extent to which specific teaching methods helped male students develop as readers. Two intact level-one reading classes, totaling 60 students, were selected through a straightforward sampling procedure and assigned to serve as the experimental and control groups. The quasi-experimental design allowed for a systematic comparison between the two groups.

As part of the intervention, the experimental group was taught through interactive reading activities, while the control group received conventional instruction. Participants were homogeneous across several characteristics: gender (all male), high-school background (science stream), age (18–19), first language (Arabic), prior English instruction (seven years), academic level (level 1), placement-test band (pre-intermediate), and course (Reading Skills).

This study received full ethical approval from the Najran University Research Ethics Committee (Approval No.: 202505-076-032278-073379) on 27/05/2025. The approval confirmed that the project, “Investigating ChatGPT-mediated mind mapping to facilitate EFL learners reading comprehension,” met the requirements of the National Committee for Bioethics at King Abdulaziz City for Science and Technology. In accordance with this approval, written informed consent was obtained from all participants before their involvement in the study. All data were anonymized to protect participant confidentiality, and the research was conducted strictly in accordance with the approved proposal, adhering to all standard conditions set by the ethics committee throughout the investigation. The results are expected to have implications for curriculum development and teaching strategies by offering insights into successful reading instruction strategies for students in the preparatory year. The program and data collection started right after obtaining ethical approval. The study started on 28/5/2025 and ended on 14/8/2025.

Moreover, this research utilized generative AI (Copilot) to assist with the search for authentic academic sources, editing, and proofreading. It was also used to rephrase and organize ideas and draft and format sections such as results and references.

### Tools

Data were collected through two instruments: an RC achievement test and semi-structured interviews. The achievement test measured participants’ RC performance before and after instruction in the ChatGPT-mediated mind mapping strategy. The interviews were used to explore participants’ learning experiences, with particular attention to the difficulties they encountered when working through reading passages using ChatGPT-assisted mind maps.

#### RC Test.

The RC test used to evaluate first-level EFL learners’ achievement in RC is recognized for its high validity and reliability. Created by experienced applied linguistics specialists with extensive expertise in teaching reading courses, the test items were meticulously selected from a question bank. A professional committee assessed the test for difficulty levels, item discrimination, and alignment with key curriculum skills such as predicting outcomes, scanning and skimming, making inferences, vocabulary acquisition, topic identification, recognizing main ideas and supporting details, and understanding paragraphs.

The RC test comprised both essay and multiple-choice questions based on two reading passages, administered within a one-hour time frame. This format offered a balanced evaluation of diverse reading skills, with multiple-choice questions assessing scanning, skimming, and identification of main ideas, and essay questions measuring inferencing and synthesis abilities. A detailed scoring rubric distributed points across four areas: comprehension (10 points), accuracy of responses (5 points), relevance to primary themes (5 points), and logical inference (5 points), yielding a total possible score of 25. Scores were categorized as follows: below 12 = weak, 12–17 = average, 18–20 = good, and 21–25 = excellent.

To establish reliability, a pilot test with a sample of 30 EFL students was conducted, resulting in a Kuder-Richardson-20 reliability coefficient of 0.79. Item analysis and expert reviews further confirmed the test’s validity, ensuring it effectively measured the RC skills relevant to the study. Administered in a controlled classroom environment, the RC test was standardized with consistent instructions and supervision to minimize external influences. A pre-test was conducted before the intervention to establish baseline RC abilities across groups, with results indicating no significant differences between groups in their initial performance. These details underscore the RC test’s rigor, robustness, and suitability as a standardized measure for assessing EFL learners’ RC skills within the ChatGPT-mediated mind mapping intervention.

#### Interview.

Following their consent to participate, a purposive sample of seven individuals chosen from the experimental group were interviewed in a semi-structured manner to gather in-depth insights into their learning experiences. The selection was based on several criteria intended to ensure variation and to identify information-rich cases, including active participation and interaction during the intervention, achievement scores, willingness and consent to participate, fluency in English, ability to use GPT effectively to create mind maps, and clear understanding of the role of GPT in supporting their RC. The interviews were conducted online via Zoom once the post-test had been administered. Each participant took part in a one-on-one session with the researcher, and every session was recorded; interviews lasted approximately five minutes on average.

The questions examined how effectively ChatGPT-mediated mind mapping supported RC of reading passages. They probed the perceived benefits of the approach, including its capacity to deepen understanding of the content, and—by attending to limitations and difficulties in implementation—sought to identify the obstacles students encountered while working with ChatGPT-mediated mind mapping. The questions also asked how participants saw themselves integrating the method into their own study practices and whether they intended to continue using it in subsequent courses.

The interview questions included:

1. Do you think that ChatGPT-mediated Mind Mapping was useful in comprehending reading passages? In what ways?
2. Did you face any obstacles using ChatGPT-mediated Mind Mapping while comprehending reading passages? If yes, please specify these difficulties.
3. Would you use ChatGPT-mediated Mind Mapping in your future courses? If yes, how do you think you will use it?

To establish the validity of the interview prompts, three faculty members with expertise in EFL teaching and learning—particularly in the use of technology in EFL settings—reviewed the questions in detail. They approved the final prompts, confirming both their linguistic clarity and their suitability for generating data relevant to the research questions.

Each interview was recorded using Zoom’s audio-visual features and transcribed for close examination. The transcripts were then analyzed through thematic coding, which was used to identify recurring patterns and to draw out insights from the participants’ responses.

Ethical guidelines were followed throughout the interview procedure, and participants were informed of the confidentiality protocols in place and offered the freedom to leave the study at any moment without facing any consequences.

### Intervention

The 10-week training program using ChatGPT (free version) is designed to enhance RC skills among EFL learners by integrating mind-mapping techniques with GPT support. The program is structured around explicit instruction in RC strategies—summarizing, predicting, and questioning—so that students can actively monitor and strengthen their understanding of texts. It opens with a two-hour training session, led by an experienced EFL instructor, that is designed to prepare students to use ChatGPT’s features effectively for mind mapping.

The free version of ChatGPT was chosen because its role in this intervention was not to generate visually polished mind maps, but to help learners analyze reading passages—identifying the main idea, supporting details, and additional information—and to arrange these elements into a simple mind-map layout. The free plan suits this purpose well: it allows users to work with uploaded files, summarize texts, and receive explanations in clear and accessible language, all of which align closely with the reading-comprehension aims of the intervention.

Specialized mind-mapping platforms such as MindMeister, Coggle, Xmind, and Lucidchart, by contrast, are built primarily for the visual creation, editing, and sharing of diagrams. Their main strengths lie in layout design, templates, collaboration, and export features, rather than in guiding learners through the cognitive work of interpreting and simplifying reading passages. Their free tiers are also typically limited, often capping the number of maps or diagrams a user can create, or restricting AI-supported functions to trial access.

The 10-week duration adopted for this ChatGPT-mediated RC program is consistent with earlier research on effective training periods for RC and related cognitive skills in educational settings. Adel Baghagho et al. [7], among others, argue that sustained training of 6–12 weeks gives students enough time to internalize and apply new strategies—digital mind mapping included—without overloading their schedules, and report that mind mapping delivered over such a structured period improved EFL students’ comprehension by organizing textual information visually and fostering deeper engagement with the text. Aljaser [8] and Almelhi [9] reach a similar conclusion: gradual, week-by-week work with electronic mind maps was associated not only with better comprehension but also with more positive attitudes toward learning English, suggesting that consistent, extended practice yields the strongest cognitive gains. Alomari and Alhorani [11] demonstrate that intervals of at least eight weeks are particularly effective for RC improvements in EFL settings, providing students with enough time to familiarize themselves with mind mapping techniques and integrate them into their learning processes. Integrating ChatGPT over a structured period offers additional advantages by combining technology with traditional methods. The growing interest in AI in education, as examined by Xu et al. [36] and Limna et al. [37], points to AI tools’ ability to promote personalized learning, which can be a novel addition to face-to-face instruction. This integration into a 10-week program thus capitalizes on the optimal training period for critical thinking skills and represents an innovative approach to RC in educational technology.

Throughout the program, students engage in hands-on training sessions, where they practice applying mind mapping to identify main ideas, supporting details, and relevant information in reading passages, guided by the prescribed reading textbook: *Academic Progress Reading & Writing Student Book (P2)*. After the initial two-hour orientation session in Week 1, the experimental group completed one GPT-mediated mind-mapping task each week during the regular reading class. Each task was carried out within one class session of approximately [30 minutes], and the activity followed the same instructional sequence across the 10-week intervention.

The program builds in follow-up activities and regular reinforcement across the study period, giving students repeated opportunities to consolidate each RC strategy. A pre-test established baseline performance for both groups, after which a post-test and semi-structured interviews were used to evaluate the program’s effect on students’ comprehension and on their experiences of using GPT for mind mapping. Taken together, the design offers a manageable yet intensive framework for integrating technology into EFL reading instruction, in line with current research on strategy training in language education.

In the first week of the semester, an experienced EFL instructor with a background in technology integration led a two-hour classroom training session. Its aim was to equip students with the skills needed to use ChatGPT’s features to apply the mind-mapping technique successfully. This orientation included: (a) a teacher demonstration of how to read a passage first and identify the main idea, supporting details, and additional information; (b) guided practice in entering prompts into ChatGPT; (c) explanation of how to review and validate AI-generated output; and (d) a short practice task completed individually or in pairs. A full sample training lesson, worksheet, and sample prompts are provided in S1 Appendix.

The independent variable examined here is the instruction on strategy use (GPT-mediated mind mapping technique), based on the hypothesis that explicit use of mind mapping mediated by GPT will impact students’ RC. Following Oxford’s [38] model of strategy training, the program involved steps such as assessing learners’ needs and resources, assigning strategies, considering anticipated benefits and motivation, preparing materials and activities, implementing training, and evaluating its effectiveness.

To determine baseline RC performance, a pre-test was taken by the experimental and control groups before the intervention. To guarantee that the two groups performed equally, the pre-test was given using the identical RC accomplishment test as the post-test. The pre-test covered the same material as the post-test and had two open-ended and three multiple-choice questions. The experimental and control groups’ RC skills were equivalent before the intervention, as evidenced by the results, which revealed no discernible differences.

After the pre-test, the training program for the experimental group commenced. The experimental group received training on mind mapping techniques using tasks and activities from the textbook, integrating identified strategies. The teacher-trainer guided students using mind mapping with ChatGPT to understand reading passages by identifying main ideas, supporting details, and additional information.

The experimental group received the necessary training on how to use GPT-mediated techniques to support RC. After this training, learners used ChatGPT to help them identify the main idea, supporting details, and additional information in reading passages and to organize these elements into simple mind maps. In contrast, the control group was taught the same reading-comprehension objectives through conventional mind-mapping instruction, using traditional classroom methods without the support of any generative AI tools. Thus, both groups were exposed to mind mapping as a comprehension strategy, but only the experimental group used ChatGPT as a mediating tool in the process.

During training, the experimental group used GPT to construct mind maps of the prescribed reading passages. The training focused on how to build mind maps that, by drawing on GPT’s features, would deepen students’ comprehension of the text. Through group activities and hands-on practice, students engaged directly with the technology during each session. Using ChatGPT, they learned to rely on a mind map to identify the key points and illustrative features of a sample reading passage drawn from the required textbook: *Academic Progress Reading & Writing Student Book (P2)*. The procedure was built around co-construction rather than passive acceptance of the AI’s output. In each task, students first read the assigned passage on their own and jotted down what they took to be the main idea and the key details. They then submitted a guided prompt to ChatGPT to obtain a preliminary text-based mind map. Once the response appeared, they compared it against the original passage, verified whether the main idea, supporting details, and additional information had been captured accurately, and either refined the prompt or edited the mind map by hand. In this sense, ChatGPT functioned as a mediating support tool, while the final version of the map remained subject to student review and teacher confirmation.

In this intervention, ChatGPT is used only to support RC. After reading a passage, learners use ChatGPT to create a simple mind map that identifies the main idea, the supporting details, and any additional information in the text. This helps learners organize what they read in a clear visual form and improves their understanding of the passage. The reading texts used for this purpose can include topics such as hobbies, favorite pastimes, start-up companies, world records, social media, deep-sea exploration, and deep-sea creatures.

The prompt pattern taught to students was intentionally simple and consistent. Students were trained to use prompts such as: “Help me understand this reading passage. Identify the main idea, three supporting details, and two pieces of additional information. Then make a simple mind map in easy English,” and “Turn this reading passage into a simple mind map. Put the main idea in the center, add branches for supporting details, and add one branch for additional information. Use short phrases only.” These prompt models were repeated throughout the intervention so that students learned to use ChatGPT as a structured reading-comprehension aid rather than as an automatic answer generator. Table 1 presents sample mind maps generated by students using these prompts across a range of reading passages from the course textbook, with additional examples provided in S1 Appendix.

**Table 1:** Sample RC mind maps generated with ChatGPT.

No.	Reading Passage/ Topic	Main Idea (Center of the Mind Map)	Supporting Details	Additional Information
1	Hobby/ Bird Watching	A hobby can be interesting and enjoyable.	Place: mountains; equipment: binoculars, camera; frequency: every month.	Favorite bird: eagle; activities: climbing, taking photos; reason: exciting.
2	Favorite Pastimes in MENA	People in the MENA region have different favorite pastimes.	Family time: about 40%; Internet: about 30%; some spend 1 hour online, others 5 hours.	Survey year: 2013; over 10,000 people answered the survey.
3	Start-Up Company/ Careem	A regional company grew from a small start-up into a major business.	Founded in 2012; service: taxi app; based in Dubai; active in many cities.	Bought by a parent company in 2019; 30 million+ users; 1 million+ drivers; 15 + countries.
4	World Record/ Skydiving	A skydiver broke important world records.	Height: 39 km; speed: 1,340 km/h; longest free fall; fastest speed.	Date: 14 October 2012; free fall lasted more than 4 minutes; two world records were broken.
5	Sports Profile/ Famous Athlete	A famous athlete achieved success through talent and hard work.	Occupation: footballer; clubs played for; important records; national team.	Date of birth; country; award received; daily routine such as training and gym.
6	Social Media Usage in MENA	Social media is widely used in the MENA region.	Facebook: 164 million users; YouTube channels increased; many young Arabs get news online.	Egypt has very high Facebook use; Snapchat is widely used in the GCC and KSA.
7	Social Networking: Pros and Cons	Social networking has both advantages and disadvantages.	Pros: communication, jobs, education, volunteering; Cons: security, cyberbullying, isolation, health concerns.	Extra examples: privacy problems, lower grades, keeping in touch with family and friends.
8	Deep-Sea Exploration	Scientists explore the deep sea to learn more about life there.	A deep trench was explored; a special submarine was used; samples, photos, and videos were collected.	Conditions include no sunlight and very high pressure; more expeditions are planned; there may be new species.
9	Deep-Sea Creatures	Deep-sea animals have special adaptations that help them survive.	Large eyes; strong senses; slow metabolism; small bodies; ability to save energy.	The deep sea has little food and oxygen; some animals produce light.
10	Two Creative Arabs	Two successful Arab figures are creative in different fields.	One works in government and photography/poetry; one works in fashion design; both are successful.	Achievements include awards, global recognition, and professional success in different countries.
11	Two Adventurous Arabs	Two Arab adventurers achieved success in mountain climbing.	One climbed the Seven Summits; the other completed major exploration challenges.	One also promotes climbing in Saudi Arabia; the other is an entrepreneur and inspirational speaker.
12	Food from Around the World	Different countries have typical dishes with special ingredients and cooking methods.	Dishes come from different countries; each one has ingredients, preparation steps, cooking methods, and serving style.	Examples include local and international foods, festival dishes, and recipe stages.

To make validation explicit, students were instructed to check each AI-generated mind map against the original passage using questions (see S1 Appendix). If any part was incomplete, inaccurate, or oversimplified, students revised the output either by re-prompting ChatGPT or by editing the map themselves. The teacher monitored this process and confirmed the final version during classroom discussion.

Regular follow-up activities were planned to reinforce the methods gained over the 10-week study period. Thus, each instructional cycle involved reading the passage, prompting ChatGPT, reviewing the generated output, validating it against the source text, and revising the final mind map. A post-test (standardized mid-term test) was given at the end of the training session to assess RC’s progress. This evaluation functioned as a quantifiable gauge of program efficacy and individual development. Students then participated in semi-structured interviews to discuss their experiences utilizing mind maps mediated by GPT to help them understand reading passages.

### Data analysis

To investigate the first research question, both descriptive and inferential statistical analyses were conducted using SPSS version 28. The researcher began by calculating descriptive statistics (means, standard deviations, medians, ranges, and interquartile ranges) for both groups’ pre-test and post-test scores. The normality of distribution was assessed using the Shapiro-Wilk test and Kolmogorov-Smirnov test with Lilliefors correction, as recommended for sample sizes under 50. Homogeneity of variances was evaluated using Levene’s test for equality of variances. Given that the experimental group’s post-test scores violated the assumption of normality (Shapiro-Wilk p = .001), the non-parametric Mann-Whitney U test was employed to compare the post-test scores between groups, rather than the independent samples t-test. For the pre-test comparison, where both groups showed normal distribution, an independent samples t-test was appropriately used to establish baseline equivalence. The reliability of the RC test was assessed using the Kuder-Richardson Formula 20 (KR-20), which yielded a coefficient of 0.79, indicating acceptable internal consistency for the measurement instrument. The practical significance of the intervention was evaluated by calculating the effect size (r) using the formula r = Z/√N, where Z is the standardized test statistic from the Mann-Whitney U test and N is the total sample size.

**Fig 2:**
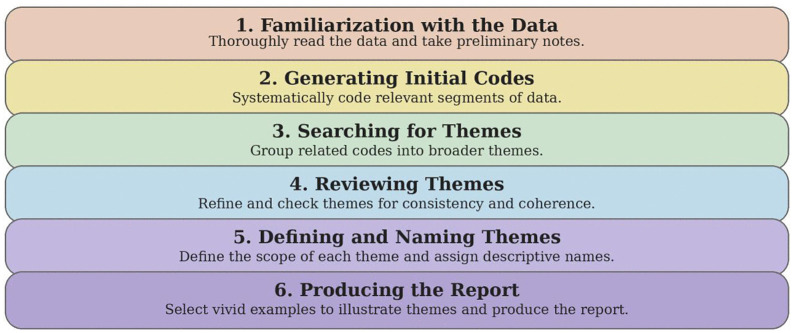
Braun and Clarke’s [39] six-phase model for thematic analysis.

For the second research question, which explored participants’ learning experiences with ChatGPT for RC, the researcher conducted semi-structured interviews. The researcher developed interview protocols to guide participants in reflecting on their experiences using the ChatGPT-mediated mind map technique to comprehend reading passages in an EFL context. Participants discussed challenges they encountered when using GPT for learning RC through mind maps. Qualitative data from these interviews underwent thematic analysis, with researcher categorizing responses based on recurring themes [39]. Fig 2 illustrates Braun and Clarke’s [39] six-phase model for thematic analysis. Each phase includes a brief description to guide the systematic process of identifying and defining themes within qualitative data. This model emphasizes a structured approach to ensure in-depth and reliable insights from qualitative studies.

## Results

### Role of ChatGPT-mediated mind mapping technique in enhancing EFL readers’ RC

This report presents a complete statistical analysis of an experimental study investigating the effects of a ChatGPT-mediated mind mapping technique on the RC of EFL learners. The analysis includes pre-test equivalence testing, normality assessments, and both descriptive and inferential analyses of post-test results to provide a comprehensive understanding of the intervention’s effectiveness.

#### Pre-Test analysis: Establishing group equivalence.

The pre-test descriptive statistics in Table 2 reveal important initial characteristics of both groups. While the measures of central tendency (mean and median) are remarkably similar between groups, indicating comparable average performance levels, the control group demonstrates substantially greater variability in scores. This is evidenced by the larger standard deviation (4.05 vs 2.58), variance (16.43 vs 6.64), and interquartile range (5.62 vs 2.50). The Experimental group’s scores were more tightly clustered around the mean, while the control group showed more dispersed performance levels.

**Table 2:** Descriptive statistics for experimental and control groups (pre-test).

Measure	Experimental Group	Control Group
Sample Size (n)	30	30
Mean (Average)	17.72	17.02
Median	17.50	17.25
Standard Deviation	2.58	4.05
Variance	6.64	16.43
Range	10.5	13.5
1st Quartile (Q1)	16.50	14.88
3rd Quartile (Q3)	19.00	20.50
Interquartile Range (IQR)	2.50	5.62

As shown in Table 3, both normality tests (Kolmogorov-Smirnov and Shapiro-Wilk) yielded non-significant results (p > .05) for both groups, confirming that the pre-test RC scores followed a normal distribution. This satisfies the normality assumption for parametric testing of pre-test data.

**Table 3:** Tests of normality (pre-test).

Variable	Group	Kolmogorov-Smirnov		Shapiro-Wilk	
		Statistic	Sig.	Statistic	Sig.
RC Scores	Experimental	0.096	.200	0.971	.567
RC Scores	Control	0.092	.200	0.963	.364

In Table 4, the independent samples t-test confirms no statistically significant difference between groups at baseline (t(58) = 0.78, p = 0.437). This establishes that any post-intervention differences cannot be attributed to pre-existing differences in RC ability.

**Table 4:** Independent samples t-test for pre-test RC scores.

Group	N	Mean	Std. Deviation	t	df	Sig. (2-tailed)
Experimental	30	17.72	2.58	0.78	58	0.437
Control	30	17.02	4.05			

### Post-test analysis: Evaluating intervention effects

As depicted in Table 5, the post-test descriptive statistics reveal substantial differences between groups. The experimental group shows markedly higher central tendency across all measures (means, medians, & modes). Notably, while the Experimental group maintained relatively consistent performance (SD = 2.77), the control group continued to show greater score dispersion (SD = 4.22). The experimental group’s higher minimum score (16.5 vs 10) suggests more consistently strong performance across all participants.

**Table 5:** Descriptive statistics for experimental and control groups (post-test).

Measure	Experimental Group	Control Group
Sample Size (n)	30	30
Mean (Average)	21.33	17.04
Median	22.50	17.25
Standard Deviation	2.77	4.22
Variance	7.70	17.80
Range	8.00	14.00
Minimum	16.50	10.00
Maximum	24.50	24.00

Table 6 displays that the normality tests indicate violation of the normality assumption for the Experimental group (Shapiro-Wilk p = .001) while the control group maintained normal distribution (p = .364). This violation necessitates the use of non-parametric tests for post-test comparisons.

**Table 6:** Tests of normality (post-test).

Variable	Group	Kolmogorov-Smirnov		Shapiro-Wilk	
		Statistic	Sig.	Statistic	Sig.
RC Scores	Experimental	0.253	.035	0.858	.001
RC Scores	Control	0.092	.941	0.963	.364

As shown in Table 7, the Mann-Whitney U test identified a statistically significant difference in post-test RC scores between the two groups (U = 180.00, p < .001). The experimental group (Mean Rank = 39.50) clearly outperformed the control group (Mean Rank = 21.50). With an effect size of r = 0.52, the result carries medium-to-large practical significance, indicating that the intervention was educationally as well as statistically meaningful.

**Table 7:** Mann-Whitney U test for post-test RC scores.

Group	N	Mean Rank	Mann-Whitney U	Z	Asymp. Sig. (2-tailed)	Effect Size (r)
Experimental	30	39.50	180.00	−3.99	<.001	0.52
Control	30	21.50				

Taken together, the statistical analyses offer strong evidence that ChatGPT-mediated mind mapping supported EFL RC in this study. The pre-test analysis confirmed that the two groups entered the study with comparable RC ability, ruling out pre-existing differences as an alternative explanation for what followed. At post-test, the experimental group scored substantially higher than the control group and, as reflected in the smaller variability of their scores, showed more uniform gains across participants. Combined with a significant Mann-Whitney U result (p < .001) and a substantial effect size (r = 0.52), these patterns provide solid evidence that was linked to improved RC performance. The uniformity of the effect observed within the experimental group further suggests that the intervention may have benefited a broad range of learners rather than only the higher-achieving ones—a point worth underlining given the control group’s persistent variability throughout the study. In practical terms, the 4.29-point mean difference represents a sizeable shift, moving the experimental group from moderate to high performance levels while the control group showed only minimal gain over time.

Overall, the post-test findings lend support to ChatGPT-mediated mind mapping as an instructional approach for EFL RC. Students in the experimental group performed significantly better than their counterparts in the control group, and the size of this difference was educationally meaningful. However, given the quasi-experimental nature of the study, these findings should be interpreted as evidence supporting the usefulness of the approach, rather than as definitive proof of causation.

### EFL students’ learning experiences of utilizing the GPT-mediated mind mapping technique in RC passages

Qualitative data from the semi-structured interviews were analyzed thematically following Braun and Clarke’s [39] six-phase model, which involves familiarization with the data, generating initial codes, searching for themes, reviewing themes, defining and naming themes, and producing the report. This systematic procedure enabled the researcher to identify recurring patterns in participants’ responses and to organize them into coherent themes and subthemes. The thematic analysis of the interview data revealed three major themes related to students’ experiences with GPT-mediated mind mapping in RC. These themes, along with their associated subthemes, are presented in Table 8.

**Table 8:** Emerging themes and subthemes from the qualitative interview data.

Major Theme	Subthemes/ Topics
Perceived Benefits of GPT-Mediated Mind Mapping	Breaking down complex ideas
	Visualizing relationships between concepts
	Enhancing understanding and comprehension
	Improving memory retention
	Facilitating summarization of reading passages
	Increasing engagement in studying and review
Perceived Difficulties in Using GPT-Mediated Mind Mapping	Accuracy-related issues
	Difficulty understanding complex content
	Limitations in usability and flexibility
	Lack of access to devices
	Internet connectivity problems
	Time and effort required to use the tool effectively
Future Use and Perceived Scope of Application	Organizing thoughts and ideas
	Summarizing complex topics
	Creating study guides
	Brainstorming and linking ideas
	Outlining essays and reports
	Supporting collaboration in group projects
	Enhancing visual learning

#### Benefits.

Based on the responses provided by students regarding the usefulness of ChatGPT-mediated Mind Mapping in comprehending reading passages, the participants’ responses indicate that students found ChatGPT-mediated Mind Mapping useful primarily for breaking down complex ideas. S1 added, “Certainly, it assisted me in simplifying complex ideas.” S2 said, “Mind maps effectively elucidated connections between various concepts.” S3 answered, “ChatGPT-mediated mind mapping clearly emphasized crucial information.” In addition, the analysis shows that some participants used ChatGPT for visualizing relationships between concepts. S4 told, “It facilitated identifying the main points and their connections.” S5 narrated, “Visualizing details aided in better retention.” S6 commented, “Mind mapping improved my understanding of the text’s structure.” Moreover, other participants’ answers reveal that they used ChatGPT for enhancing understanding and memory retention. S7 provided, “Using ChatGPT for mind mapping enhanced my overall comprehension.” S8 said, “Mind maps proved effective in summarizing passages.” S9 answered, “It prompted deeper contemplation of the content.” Finally, a participant used ChatGPT for making studying more engaging and memorizing. S10 added, “It enhanced the engagement of studying and reviewing passages.”

#### Difficulties.

Based on the responses provided by students regarding difficulties when using ChatGPT-mediated Mind Mapping in comprehending reading passages and difficulties related to technology availability, time, and effort, the analysis shows that some students encountered challenges with accuracy. S1 said, “Facilitated connections, though occasionally the layout was confusing.” S2 provided, “Effective for summarizing, yet not always comprehensive.” S3 told, “Aided in grasping main ideas, but occasionally overlooked details.” S4 added, “Assisted in review, yet prioritization of key points was inconsistent.” S5 narrated, “Useful for brainstorming, though comprehension gaps were evident.” S6 commented, “Helped organize thoughts, yet desired more flexibility for adjustments.” In addition, some other participants showed difficulties understanding complex content, and the usability of ChatGPT-mediated Mind Mapping in comprehending reading passages. S7 answered, “Helped organize ideas, but sometimes missed important details.” S8 added, “Made grasping main points quicker, but struggled with harder parts.” And S9 told “Improved seeing the whole story but didn’t always understand every word.” S10 said, “Simplified understanding, but occasionally made things too simple.”

Moreover, they faced issues related to technology availability. S1 said, “Couldn’t use it sometimes because I didn’t have a computer.” S2 added another difficulty, “Hard to start my work without a computer.” S3 narrated, “Couldn’t finish my assignments without a computer.” Furthermore, they encountered issues related to internet connectivity. S4 commented, “My internet was slow, so it took longer to finish.” S5 answered, “Slow internet made it frustrating to complete tasks.” S6 provided, “It was hard to access and use effectively with my slow internet.”

Finally, they faced issues concerning the time and effort required to effectively utilize the tool. S7 said, “It was difficult to find time with my busy schedule.” S8 added, “I had a hard time balancing it with my other responsibilities.” S9 answered, “Setting it up and using it properly required a lot of effort.” S10 told, “Understanding and using the program took a lot of effort.”

#### Future work.

Based on the students’ responses regarding whether they would use ChatGPT-mediated Mind Mapping in their future courses and how they intend to use it, the responses indicate that students are inclined to use ChatGPT-mediated Mind Mapping for various purposes such as organizing thoughts. S1 said, “Yes, it helps me sort out my thoughts clearly.” S2 added, “Yes, I’ll use it to make study guides for exams.” S3 answered, “I intend to use it to plan out essays and reports.” Other answers provided that they used mind maps via ChatGPT for summarizing complex topics. S4 said, “Absolutely, it’s excellent for summarizing complex topics.” S5 added, “Yes, it makes studying more fun and visual.” S6 concluded, “Yes, it saves time when going through lots of information.” Moreover, some other participants commented that they would use it for brainstorming and connecting ideas, creating study guides, outlining essays and reports, collaborating on group projects. S7 said, “I would use it to think of ideas for projects.” S8 added, “Yes, it’s helpful for working together on group projects.” S9 thought, “Definitely, it’s good for linking ideas together.” Finally, one student thought that mind maps would enhance visual learning experiences. S10 commented, “It’s useful for people who learn best visually, like me.”

## Discussion

The findings of the present study provide converging quantitative and qualitative evidence supporting the potential value of the GPT-mediated mind mapping technique in enhancing students’ RC. The quantitative results revealed a significant difference in RC scores between the experimental group, which utilized the GPT-mediated mind mapping technique, and the control group. The effect size results further support this finding by indicating a meaningful difference in RC scores in favor of the experimental group. Taken together, these findings suggest that the GPT-mediated mind mapping technique may support students’ RC performance. The qualitative findings reinforce this result, as the interviewed students reported that ChatGPT-mediated mind mapping helped them break down complex ideas, visualize relationships between concepts, enhance understanding, retain information more effectively, and summarize passages more clearly. This alignment between the two sets of findings suggests that the statistical improvement in the experimental group was accompanied by clear learner perceptions of improved comprehension processes.

The interpretation of these results is strengthened by several considerations. Firstly, rigorous statistical analyses such as the Mann-Whitney U test demonstrated significant differences in RC scores between the experimental and control groups. This statistical significance suggests that the observed differences in RC scores are unlikely to be due to random chance. Secondly, the substantial effect size of the training program underscores the practical significance of the intervention. Effect size measures the magnitude of the difference between groups, independent of sample size, and the findings indicate a medium-to-large effect. This suggests that the observed difference in RC scores may be associated with the GPT-mediated mind mapping technique, highlighting its possible contribution to improved RC performance in the experimental group. Thirdly, the consistency of results across the non-parametric (Mann-Whitney U) test further bolsters the reliability of the findings. These analyses consistently showed that the experimental group, utilizing the GPT-mediated mind mapping technique, achieved higher RC scores compared to the control group. Importantly, the qualitative findings help explain this improvement: students stated that the technique allowed them to reorganize passage content visually, identify the main points more easily, connect supporting ideas, and study in a more engaging and meaningful way. Thus, the qualitative data strengthen the interpretation of the quantitative findings by clarifying how the intervention may have supported comprehension in practice.

The findings of the study can be interpreted most clearly through Vygotsky’s [30] Sociocultural Theory and Sweller’s [31] Cognitive Load Theory, which together provide a focused explanation for the potential value of GPT-mediated mind mapping in RC. From a sociocultural perspective, ChatGPT appears to have functioned as a mediating scaffold that supported learners while they analyzed reading passages and organized them into mind maps. Rather than working independently from the outset, students used ChatGPT to help identify the main idea, supporting details, and additional information, and then reviewed and refined the generated output. In this sense, the tool may have supported learners within their Zone of Proximal Development by helping them carry out comprehension tasks that might have been more difficult without guided assistance. This interpretation is supported by the qualitative findings, as students reported that the technique helped them simplify difficult passages, understand relationships among ideas, and organize information more clearly.

The findings may also be understood through Cognitive Load Theory. RC in a foreign language often places a heavy demand on working memory because learners must manage multiple pieces of information at the same time. By recasting reading passages into a visual structure, GPT-mediated mind mapping appears to have reduced extraneous cognitive load and freed learners to concentrate on meaning. The validation stage seems particularly important here: students had to compare the AI-generated map with the original passage, check its accuracy, and revise it where necessary—steps that are likely to have drawn their attention back to the text itself and encouraged deeper processing of the passage. The interview data are consistent with this reading; participants repeatedly noted that the technique made complex information easier to handle, easier to remember, and easier to summarize. Taken together, the two theoretical perspectives suggest that the educational value of GPT-mediated mind mapping lies in pairing guided mediation with clearer organization of textual information, so that comprehension can unfold within a structured and cognitively manageable framework.

Placed alongside earlier research on mind mapping techniques for improving students’ RC, the present findings reveal both continuities and distinctive features. The study documents a substantial rise in RC scores among students using GPT-mediated mind mapping relative to those in the control group, a result that echoes a considerable body of work reporting positive effects of mind mapping on RC across a wide range of educational contexts and student populations [6–22]. The qualitative findings of the present study further strengthen this similarity, since students also expressed positive perceptions of the technique and emphasized its usefulness for understanding, organizing, and remembering textual content. In addition, both current and previous studies often cite theoretical frameworks rooted in cognitive theories of learning and memory. These theories suggest that visually organizing and structuring information can significantly enhance comprehension and retention, providing a solid theoretical basis for the effectiveness of mind mapping in improving RC skills.

However, studies diverge in their technological approaches and specific outcomes measured. Previous research has explored a range of electronic mind mapping tools and platforms, such as Edmodo and specific software applications tailored for educational purposes [6–22]. In contrast, the current study distinguishes itself by utilizing GPT (Generative Pre-trained Transformer) technology for mind mapping, thereby showcasing advancements in AI-driven educational tools. Moreover, the specific focuses and outcomes vary among studies. While some investigations emphasize specific facets of RC enhancement, such as summarization skills or critical thinking abilities, the current study primarily assesses overall RC performance. The qualitative findings add a distinctive dimension to this contribution by showing that learners perceive GPT not only as a tool for visual organization, but also as a support mechanism that helps them interact with the text more actively and meaningfully. In this respect, the present study extends earlier work by highlighting not only the performance outcomes of mind mapping, but also the mediating role of GPT in shaping the learning process itself. This variation provides complementary insights into the multifaceted benefits of mind mapping techniques in educational settings.

The present findings are also consistent with recent research on the cognitive and affective dimensions of AI-supported EFL learning. The reported benefits of GPT-mediated mind mapping—such as clearer organization of ideas, deeper analysis, and more engaging study experiences—are comparable to Liu and Wang’s [32] finding that AI-supported instruction can strengthen higher-order learning processes. Likewise, students’ positive views of future use and their reports of engagement align with Wu et al. [33], who found that positive attitudes and ease of use are central to learners’ intention to adopt AI. At the same time, the challenges related to usability, effort, and access resonate with work by Derakhshan and Park [34,40], which highlights both the emotional benefits of AI-mediated instruction and the frustration or unmet needs that may arise when learners face technological or contextual barriers.

The interview on EFL readers’ learning experiences of utilizing the GPT-mediated mind mapping technique to comprehend reading passages revolved around three main themes: benefits, challenges, and future scope. Concerning benefits, the responses highlight that students find ChatGPT-mediated Mind Mapping beneficial for several reasons: it helps break down complex ideas, visualize relationships between concepts, enhance understanding and memory retention, and make studying more engaging. Moreover, this approach encourages deeper content analysis and aids in effectively summarizing passages. Overall, students view ChatGPT-mediated Mind Mapping as a valuable tool for enhancing comprehension and study efficiency when dealing with reading passages. Taken together, these reported benefits suggest that GPT-mediated mind mapping served as more than a visual aid; it acted as a comprehension scaffold that helped learners work through texts in a more active and systematic way. These reported benefits sit comfortably alongside the quantitative finding that the experimental group outperformed the control group on RC.

Turning to the difficulties students reported while learning RC through GPT-mediated mind maps, the interviews point to three recurring problems: the accuracy of the AI output, the challenge of understanding dense content, and the overall usability of ChatGPT-mediated mind mapping for working through reading passages. Students also described obstacles related to technology access, unreliable internet connections, and the time and effort the tool demanded. These accounts help explain why the intervention yielded a medium rather than a larger effect, as practical and cognitive barriers of this kind are likely to have limited how much some learners could benefit from the technique. They also point to the refinements and forms of support that would be needed to make this kind of technology work well in similar educational settings. Put differently, the qualitative evidence suggests that the success of the intervention depended not only on the instructional strategy itself, but also on the conditions under which students were able to engage with the tool.

The responses also reveal that students see a wide range of uses for ChatGPT-mediated mind mapping—organizing their thinking, summarizing complex topics, brainstorming, building study guides, outlining essays and reports, collaborating on group projects, and supporting visual learning. They valued the tool’s capacity to streamline review, foster collaboration, and accommodate different learning styles, and they viewed it positively as something to be carried forward into their future academic work. On this reading, the benefits of the intervention extend beyond the immediate gains in post-test scores: students appear to treat GPT-mediated mind mapping as a transferable academic strategy with broader educational value. This forward-looking orientation is significant, as it suggests that the intervention may have shaped not only learners’ immediate performance, but also their willingness to adopt GPT-mediated mind mapping as a useful learning strategy for future academic tasks.

Taken as a whole, and notwithstanding the technological and methodological differences across individual studies, the research consistently supports mind mapping as a valuable educational tool for improving students’ RC. The present study aligns with this broader conclusion and extends it by showing that, when mind mapping is mediated by GPT, students can achieve significantly higher RC scores while also seeing the technique as helpful for organizing ideas, deepening analysis, and strengthening memory. At the same time, the challenges reported in the interviews make clear that successful implementation depends on adequate training, ongoing support, and technological readiness. Taken together, the quantitative and qualitative findings offer a coherent and triangulated account of the potential educational value of GPT-mediated mind mapping in supporting EFL learners’ RC, while the quasi-experimental design, convenience sampling, and use of intact classes warrant caution in making definitive causal claims.

## Conclusion

The present study contributes to the growing body of research on the use of innovative technologies in language education by providing evidence that GPT-mediated mind mapping may support improvements in EFL learners’ RC. The findings from both the quantitative and qualitative data indicate that this technique was associated with a medium positive effect on students’ RC performance. In addition, learners perceived GPT-mediated mind mapping as a valuable tool for supporting comprehension, particularly by helping them break down complex ideas, visualize relationships among concepts, and retain information more effectively.

These findings suggest important pedagogical implications. Educators and curriculum developers may consider integrating GPT-mediated mind mapping into instructional practices aimed at enhancing RC. However, for this integration to be effective, students should receive appropriate training and support in using GPT for educational purposes, particularly in relation to generating mind maps, interpreting content accurately, and managing technological challenges. Greater attention should also be given to designing learning activities that accommodate different learning styles and encourage deeper analysis of reading texts.

At the same time, several limitations should be considered when interpreting the findings. First, the study employed a quasi-experimental design with convenience sampling and intact classes, which means that the findings should be interpreted cautiously and do not allow for definitive causal claims. Although the results provide evidence supporting the usefulness of GPT-mediated mind mapping, other uncontrolled variables may also have influenced the outcomes. Second, the relatively limited sample size may restrict the generalizability of the findings. Third, the study was conducted at a single university in the southwest region of Saudi Arabia and involved only male students enrolled in the Preparatory Year Program. As a result, the findings may not be fully applicable to other educational settings, academic levels, or female learners.

Future studies should include larger and more diverse samples in order to address the limitation related to the relatively restricted number of participants in the present study. A broader sample would allow researchers to examine the potential usefulness of GPT-mediated mind mapping across a wider range of EFL learners and would strengthen the generalizability of the findings.

Future research should also be conducted in multiple educational institutions and contexts, since the present study was limited to one university in the southwest region of Saudi Arabia. Replicating the study in different universities, regions, or educational systems would help determine whether the findings are context-specific or transferable to other learning environments.

Another important direction for future research is to include both male and female students, as the present study focused only on male learners. Including participants of both genders would provide a more comprehensive understanding of the potential value of GPT-mediated mind mapping and would enhance the broader applicability of the results.

In addition, future studies should involve learners from different academic levels and programs, since the current study was restricted to students enrolled in the Preparatory Year Program. Investigating the use of GPT-mediated mind mapping with learners in other disciplines or at more advanced levels of study may reveal whether the usefulness of this approach varies according to academic background or stage of learning.

Finally, future research may compare the use of GPT-mediated mind mapping across learners with different levels of English proficiency. Since variation in proficiency may influence how learners interact with the technique and benefit from it, such studies would provide a deeper understanding of the conditions under which this instructional approach may be most effective.

## Supporting information

S1 Appendix Student worksheet: Using ChatGPT to create a mind map for reading comprehension. (DOCX)
