# Supplementary material for: Investigating ChatGPT-mediated mind mapping to facilitate EFL learners’ reading comprehension
Source: PLoS One. 2026 May 18;21(5):e0336185. doi: 10.1371/journal.pone.0336185 (PMC13183188; doi:10.1371/journal.pone.0336185)
Supplement: S1 Appendix — (DOCX) [file pone.0336185.s001.docx]

***S1*** **Appendix. Student Worksheet: Using ChatGPT to Create a Mind Map for Reading Comprehension**

**Student Information**

**Name:** ____________________________________________
**Date:** _____________________________________________
**Class / Section:** ____________________________________
**Reading Topic:** _____________________________________

**Title of the Activity: Using ChatGPT to Create a Mind Map for Reading Comprehension**

**Objective of the Activity**

By the end of this activity, the student should be able to:

- identify the **main idea** of a reading passage,
- identify the **supporting details**,
- identify the **additional information**, and
- organize these ideas into a simple **mind map** using ChatGPT as a support tool.

**Instructions**

1. Read the assigned passage carefully.
2. Underline the **main idea**, **important supporting details**, and **additional information**.
3. Use the suggested prompt in ChatGPT.
4. Review ChatGPT’s response carefully.
5. Compare the response with the original passage.
6. Correct any inaccurate or missing information.
7. Write the final mind map in the space provided below.

**Part One: Before Using ChatGPT**

**A. Read the passage carefully**

Read the passage once silently. Then read it again and underline:

- the **main idea**,
- the **supporting details**, and
- the **additional information**.

**B. Write your own initial answers**

**1. What is the main idea of the passage?**

______________________________________________

**2. Write two or three supporting details from the passage.**
a. ________________________________________________________________
b. ________________________________________________________________
c. ________________________________________________________________

**3. Write one or two pieces of additional information.**
a. ________________________________________________________________
b. ________________________________________________________________

**Part Two: Using ChatGPT**

**Suggested Prompt**

Copy and paste the following prompt into ChatGPT:

Plain Text

Help me understand this reading passage.

Identify:

1. the main idea,

2. 3 supporting details,

3. 2 pieces of additional information.

Then make a simple mind map in easy English.

Show more lines

**Alternative Prompt**

Plain Text

Turn this reading passage into a simple mind map.

Put the main idea in the center.

Add branches for supporting details.

Add one branch for additional information.

Use short phrases only.

Show more lines

These prompts are designed to help students use ChatGPT to analyze the passage and organize information, rather than simply copy ready-made answers.

**Part Three: ChatGPT Output**

**Write ChatGPT’s response below**

**Main Idea**

______________________________________________

**Supporting Details**

1. ______________________________________________
2. ______________________________________________
3. ______________________________________________

**Additional Information**

1. ______________________________________________
2. ______________________________________________

**Part Four: Checking the Accuracy of ChatGPT’s Response**

Read the original passage again and compare it with ChatGPT’s output.

**Put a tick (✓) in the correct box**

- Is the **main idea** correct?
  ☐ Yes  ☐ No
- Are the **supporting details** taken from the passage?
  ☐ Yes  ☐ No
- Is the **additional information** relevant?
  ☐ Yes  ☐ No
- Did ChatGPT miss any important detail?
  ☐ Yes  ☐ No
- Do you need to improve or correct the mind map?
  ☐ Yes  ☐ No

**If yes, write your corrections here**

______________________________________________

______________________________________________

______________________________________________

This step is important because ChatGPT is used in this intervention as a mediating support tool for comprehension, not as a substitute for the student’s own reading and verification.

**Part Five: Final Mind Map**

**Write your final mind map below**

**Main Idea:**

______________________________________________

**Supporting Detail 1:**

______________________________________________

**Supporting Detail 2:**

______________________________________________

**Supporting Detail 3:**

______________________________________________

**Additional Information:**

______________________________________________

______________________________________________

______________________________________________
